# Misperceiving and misreporting input quality: Implications for input use and productivity

**DOI:** 10.1016/j.jdeveco.2022.102869

**Published:** 2022-06

**Authors:** Tesfamicheal Wossen, Kibrom A. Abay, Tahirou Abdoulaye

**Affiliations:** a International Institute of Tropical Agriculture (IITA), Kenya; b International Food Policy Research Institute (IFPRI), Egypt; c International Institute of Tropical Agriculture (IITA), Mali

**Keywords:** Agricultural inputs, Misclassification, Misperception, Misreporting, Nigeria, Smallholders

## Abstract

Farmers in developing countries routinely misperceive or misreport input quality for various reasons, which introduces substantial measurement error in farm survey data. In this paper, we motivate and illustrate, both analytically and empirically, the inferential and behavioral implications of misperception and misreporting using a unique crop variety identification data from Nigeria. Using a non-parametric framework for testing the presence of measurement error, we show that crop variety misclassification in our data is mostly driven by misperception. We then demonstrate the inferential challenges of treating misperception as misreporting and vice versa. Finally, we show that misperception induces crowding-in(out) of complementary agricultural inputs but these misperception-driven input allocations may not necessarily be yield-enhancing. As such, rectifying misperception by addressing agricultural input market imperfections may improve farmers’ investment choices and productivity outcomes.

## Highlights

• We decompose the origins of crop variety misclassification into misreporting and misperception.
• Crop variety misclassification in our data is mostly driven by misperception.
• We highlight the inferential challenges of treating misperception as misreporting.
• Misperception induced input allocations are not necessarily yield-enhancing.
• Addressing seed market imperfections may improve farmers'investment choices.

## 1 Introduction

Agricultural input markets in many sub-Saharan African (SSA) countries remain imperfect, contributing to sub-optimal use of improved agricultural inputs (Ashour et al., 2019, Bold et al., 2017). In particular, information asymmetry about the quality of inputs often limits farmers’ ability to use complementary agricultural inputs consistently (Ashour et al., 2019, Bold et al., 2017, Michelson et al., 2021, Gilligan and Karachiwalla, 2019, Wossen et al., 2020). In the absence of institutional and technological instruments for quality assurance, farmers often utilize available information in local markets and other observable attributes of inputs to form subjective beliefs about the quality and variety of inputs they acquire (Ashour et al., 2019, Bold et al., 2017, Michelson et al., 2021, Berazneva et al., 2018). However, locally available information and observable attributes of inputs may not reveal the authenticity and quality of inputs accurately and hence farmers may misperceive and/or misreport true input quality. In the context of improved crop varieties, the focus of this paper, the difficulty of observing quality in the local markets is often compounded by the credence nature of seed quality: neither genetic nor physical quality traits can be observed and assessed by farmers accurately at the time of exchange (Ashour et al., 2019, Michelson et al., 2021). For instance, using genetic test based varietal identification, recent studies find that farmers in developing countries substantially misreport or misperceive the improvement status of the crop varieties they grow (Wossen et al., 2019a, Wineman et al., 2020, Floro et al., 2018, Kosmowski et al., 2016, Maredia et al., 2016).

In many developing countries, measurement error in input quality or type is a pervasive feature of farm survey data (Carletto et al., 2013, Gourlay et al., 2019, Beegle et al., 2012). Focusing on crop variety identification, this paper considers two important sources of measurement error in farm survey data: misreporting, which arises due to poor measurement (e.g., erroneous survey response or recording errors during data collection) and misperception or mistaken beliefs which arises due to various frictions such as seed market imperfections (Wilhelm, 2019, Drerup et al., 2017). In the case of misreporting, farmers’ latent perceptions are expected to be identical with the truth since farmers misreporting their true perceptions are assumed to know and base their decision up on their latent perceptions instead of its measurement (Lewbel, 2007, Chetty, 2012). On the other hand, misperception is not a survey error *per se* and refers to the deviation between farmers’ latent perceptions and the truth, an informational asymmetry that reflects the underlying decision making process of

farmers, including their input and production choices (Drerup et al., 2017). As such, while self-reported values may differ from the truth both under misreporting and misperception, farmers’ observed input use and intensification choices are expected to deviate from the frictionless optimum under misperception but not misreporting in the sense that misperception cannot be conditionally independent of farmers’ decision making behavior (Drerup et al., 2017, Hu and Wansbeek, 2017, Wilhelm, 2019, Chetty, 2012). Moreover, when the misreported or misperceived variable of interest is binary, its measurement error (i.e., misclassification) is inherently nonclassical as the true value and its measurement share the same discrete support, making the classical measurement error assumptions implausible (Mahajan, 2006, Lewbel, 2007, Hausman et al., 1998, Hu, 2008, DiTraglia and García-Jimeno, 2019, Meyer and Mittag, 2017).^1^ Therefore, understanding the inferential and behavioral implications of misclassification requires identifying its origins: survey misreporting or misperception.

Using a unique self-reported and genetic-test based cassava variety identification data from Nigeria, this paper aims to identify the origins of variety misclassification based on the informational content of farmers’ complementary input choices (i.e., fertilizer and herbicide use in our context). Variety misclassification in this paper specifically refers to whether a farmer knows/believes that he/she is growing an improved or local variety, which can be driven by either misreporting (i.e., the farmer knows the true genetic quality of the variety but misreport it in the survey) or misperception (i.e., the farmer holds mistaken beliefs about the true genetic quality of the variety).^2^ In the context of Nigeria, cassava variety misperception is related, at least partially, to the informality of the underlying cassava seed market. In particular, due to the credence nature of seed quality, farmers may acquire planting material that is not what they think it is due to asymmetric quality information either because sellers have lost track of the variety type being exchanged, or through willful deception (Wineman et al., 2020, Wossen et al., 2020, Jaleta et al., 2020). We focus on crop variety misperception since accurate knowledge of variety traits affect the adoption decision of complementary farm inputs for improved seed (Emerick et al., 2016, Macours, 2019). For instance, Emerick et al. (2016) find evidence of crowding-in of fertilizer and other modern cultivation practices among adopters of improved rice varieties in India. In the context of Tanzania, Wineman et al. (2020) find that farmers apply more fertilizer when they perceive (correctly or incorrectly) growing an improved maize variety. Similarly, Ashour et al. (2019) find that the supply of adulterated and substandard agricultural inputs in local markets affect farmers’ perception about input qualities and willingness to adopt modern inputs.

Against this backdrop, this paper aims to examine the following inferential and behavioral implications of crop variety misclassification. First, building on the existing measurement error literature (Mahajan, 2006, Wilhelm, 2019), we identify and decompose the origins of variety misclassification into misreporting and misperception. We do so in a data-driven fashion, by examining the conditional means of input use functions non-parametrically, which are expected to vary across the two underlying sources of misclassification. Second, we examine the inferential implications of variety misclassification focusing on the estimation of input complementarity relationships. In particular, we derive and characterize the expected bias that one obtains under misperception and misreporting and show, both analytically and empirically, the inferential challenges of treating misperception as misreporting. Third, we propose appropriate empirical specifications to carry out hypothesis tests and statistical inference on input complementarity relationships in the presence of misperception and misreporting. Fourth, we empirically study the behavioral implication of misperception, focusing on farmers’ observed fertilizer and herbicide use and intensification choices. Finally, we analytically and empirically relate the implication of variety misperception and the implied input use behavior of farmers to inference related to yield.

Our non-parametric test results suggest that farmers’ latent variety perceptions are identical to their reports but not with the genetic test results, implying variety misclassification, at least in our setting, is mostly driven by misperception instead of misreporting. We then show that these misperceptions have important inferential implications, particularly for inference related to the estimation of input complementarity relationships as well as input and land productivity. More specifically, we show that treating variety misperception as misreporting results in substantially biased and spurious input complementarity relationship estimates. For instance, consistent with our non-parametric test results, we find that estimating input complementarity relationships using farmer variety reports as a proxy for their true but unobserved perceptions results in unbiased input complementarity relationship estimates. However, estimating input complementarity relationships using the genetic test results as a proxy for farmers’ unobserved variety perceptions yields input complementarity estimates that are biased downward by up to 70 percent, implying treating misperception as misreporting can lead to potentially wrong policy prescriptions.

In terms of behavioral response, we show that farmer variety perceptions (correct or incorrect) influences the allocation of fertilizer and herbicide inputs. Specifically, we find that false positive(negative) variety perceptions induce crowding-in(out) of fertilizer and herbicide inputs. However, our estimates on the yield response of these inputs suggest that misperception induced input allocations may not necessarily be yield-enhancing. That is, by distorting the allocation of complementary farm inputs, misperception could introduce inefficiencies in the production process. Besides the inferential and behavioral implications of misperception, our results underscore the potential costs of agricultural input (and information) market imperfections on farmers’ investment choices and productivity outcomes. In particular, crop variety misperception due to seed market imperfections might explain the co-existence of input over(under) use and low agricultural productivity as input complementarities are driven by farmer perceptions (correct or incorrect), irrespective of the true genetic quality of the crop varieties they grow. As such, rectifying misperception by addressing agricultural input and seed market imperfections can play an important role in improving farmers’ investment choices and productivity outcomes.

This paper broadly relates to the literature on nonclassical measurement error (Mahajan, 2006, Black et al., 2000, Frazis and Loewenstein, 2003, Lewbel, 2007, Aigner, 1973, Battistin et al., 2014, Bollinger, 1996, Schennach, 2007, Kane et al., 1999, Hu, 2008, DiTraglia and García-Jimeno, 2019) and makes a unique contribution to the evolving literature on the inferential and behavioral implications of misperception and misreporting in farm survey data (Wossen et al., 2019a, Abay et al., 2021). Our results provide a first-hand empirical evidence that measurement error in survey responses can be driven by misperception instead of misreporting, with distinct inferential and behavioral implications. Our paper also relates to recent studies uncovering substantial misallocation of agricultural inputs across plots and crops in African agriculture (Gollin and Udry, 2019, Restuccia and Santaeulalia-Llopis, 2017). Finally, this paper also contributes to the literature on the behavioral dimensions of technology adoption (Bulte et al., 2014, Macours, 2019).

The remainder of the paper is organized as follows. Section 2 describes our analytical framework to motivate and illustrate the inferential and behavioral implications of crop variety misclassification. In Sections 3, 4, we report the data, research setting and our empirical estimation strategies, respectively. Section 5 reports the main results and Section 6 provides concluding remarks.

## 2 Analytical framework

In this section, we layout an analytical framework to motivate and illustrate the inferential and behavioral implications of misclassification. Focusing on farmers’ crop variety misclassification, our analytical framework proceeds as follows: First, we characterize the expected bias under survey misreporting and misperception. We then introduce a non-parametric framework to carry out hypothesis tests for the presence of misclassification. Finally, we discuss our non-parametric and parametric estimation methods under our maintained identification assumptions.

### 2.1 Input use decision

Suppose that a farmer makes input use decision based on her perceived crop variety type ($T^{*}$) at the time of input use choices. Although our empirical tests are based on non-parametric specifications, for simplicity, we assume the following parametric specification for input use:

(1) $$X=\beta T^{*}+{ɛ}^{*}$$

where $T^{*}$ is farmers’ variety perception (i.e., whether it is improved or not) at the time of their input choices, X denotes farmers’ observed complementary input choices (e.g., fertilizer and herbicide inputs) and ${ɛ}^{*}$ is a mean-zero error term.^3^ Throughout this paper, we assume β≠0, otherwise there is no meaningful relationship to estimate and $T^{*}$ is exogenous, so that $E\left({ɛ}^{*}T^{*}\right)=0$. However, since an individual’s latent variety perception (i.e., $T^{*}$) is unobservable, inference via Eq. (1) is infeasible. Our main goal is thus to identify the conditional mean function, $\left[\beta \equiv E\left(X|T^{*}\right)\right]$, from the joint distribution of the observables in our data. Although $T^{*}$ is unobserved, we do observe two relevant proxies/surrogates in our data. The first is farmers’ self-reported variety information from the survey which we denote by $T^{R}$ (i.e., farmer reports) and the second is a result of the genetic test, which we denote by $T^{G}$ (i.e., genetic test results). Both $T^{R}$ and $T^{G}$ are binary with their support given by $\left\{0,1\right\}$. The relationship between $T^{*}$ and $T^{R}$ as well as that of $T^{*}$ and $T^{G}$ can be defined as follows:

(2a) $$T^{R}=T^{*}+\mu^{R}$$

(2b) $$T^{G}=T^{*}+\mu^{G}$$

where $\mu^{R}$ and $\mu^{G}$ are assumed to be uncorrelated with the error term of Eq. (1) (i.e., $E\left({ɛ}^{*}\mu^{R}\right)=0$; $E\left({ɛ}^{*}\mu^{G}\right)=0$, *see* Assumption 2.1 below) and $\mu^{R}$, $\mu^{G}\in \left\{-1,0,1\right\}$ depending on the value of $T^{R}$ and $T^{G}$ in relation to $T^{*}\in \left\{0,1\right\}$. In the absence of misreporting, farmer reports ($T^{R}$) are expected to be the same as their perceptions and hence misreporting arises when $T^{R}$ is a misclassified version of $T^{*}$ (i.e., $\mu^{R}\neq 0$). In our setting, $T^{*}$ could differ from $T^{R}$ due to survey errors (e.g., recording by enumerators, intentional misreporting by farmers, etc.). Similarly, the genetic test result ($T^{G}$) may differ from $T^{*}$, when farmers’ hold mistaken variety beliefs due to various market frictions. For instance, due to the informality of the underlying cassava seed market in Nigeria, farmers may hold mistaken variety beliefs due to asymmetric quality information either because sellers have lost track of the variety type being exchanged, or through willful deception (Wineman et al., 2020, Wossen et al., 2019a).

#### 2.1.1 Bias under misreporting

Suppose that the mechanism underlying variety misclassification is misreporting. In the presence of misreporting, Eq. (1) is expected to be the true data generation process (DGP) guiding farmers’ input use decision. However, the researcher observes farmer reports ($T^{R}$), which is a misclassified version of $T^{*}$. Under this scenario, the researcher operationalizes the following DGP for input use: $X=\beta^{R}T^{R}+{ɛ}^{R}$. Recalling $T^{R}=\left(T^{*}+\mu^{R}\right)$ in Eq. (2a) above, the error term of the operational model can be expressed as: ${ɛ}^{R}=\text{(}{ɛ}^{*}-\beta \mu^{R}$). To simplify our notations, let $E\left(T^{*}\right)$ and $E\left(T^{R}\right)$ be $p^{*}$ and p, respectively. Denote $\alpha_{t}=Pr\left[T^{R}=1-t|T^{*}=t\right]$, for $t\in \left\{0,1\right\}$ as the probability of false positive and negative variety reports, respectively.^4^ Given our assumption that $E\left({ɛ}^{*}\mu^{R}\right)=0$ and $E\left({ɛ}^{*}T^{*}\right)=0$, attempting to recover $\left[\beta \equiv E\left(X|T^{*}\right)\right]$ using $T^{R}$ as a measure of farmers’ true but unobserved variety perceptions ($T^{*}$) leads to the following bias:

(3) $$\begin{array}{ccc} \beta_{OLS}^{{}^{R}} & = & \frac{Cov\left(T^{R},X\right)}{Var\left(T^{R}\right)}=\frac{Cov\left(\beta T^{*}+{ɛ}^{*},T^{*}+\mu^{R}\right)}{Var\left(T^{R}\right)}\\ & = & \frac{\beta \left[Var\left(T^{*}\right)+Cov\left(T^{*},\mu^{R}\right)\right]}{Var\left(T^{R}\right)}\\ & = & \frac{\beta \left[p^{*}\left(1-p^{*}\right)\left(1-\alpha_{0}-\alpha_{1}\right)\right]}{p\left(1-p\right)}\\ & = & \frac{1}{p\left(1-p\right)}\left[\frac{\left(p-\alpha_{0}\right)\left(1-p-\alpha_{1}\right)}{1-\alpha_{0}-\alpha_{1}}\right]\beta \end{array}$$

The identity in Eq. (3) suggests that the size and sign of the bias in $\beta_{OLS}^{{}^{R}}$ depends on the extent of misreporting (i.e., $\alpha_{0};\alpha_{1}$). For instance, assuming β>0, $\beta_{OLS}^{{}^{R}}$ would be biased towards zero provided that $\alpha_{0}+\alpha_{1}<1$ and may even be estimated with the wrong sign when $\alpha_{0}+\alpha_{1}>1$. We note that, under this scenario, β can directly be identified using $T^{G}$ with the additional assumption that farmer perceptions are equal to the genetic test results (i.e., $T^{G}=T^{*}$). That is, in the absence of variety misperception, the informational content of $T^{G}$ can reveal $T^{*}$ and hence estimating Eq. (1) using $T^{G}$, instead of $T^{R}$, would overcome the bias in $\beta_{OLS}^{{}^{R}}$.

#### 2.1.2 Bias under misperception

Next, let us assume that farmer reports ($T^{R}$) are the same as their true but unobserved variety perceptions ($T^{*}$) but these reports deviate from the genetic test results (i.e., $\left(T^{*}=T^{R}\right)\neq T^{G}$). Under this scenario, the researcher who only has access to farmer reports will recover β via Eq. (3) since there is no misclassification in $T^{R}$ (i.e., $\beta_{OLS}^{{}^{R}}\equiv \beta$). Suppose that, in addition to $T^{R}$, the researcher observes $T^{G}$, which, compared to $T^{R}$, is a more reliable and accurate measure of the genetic quality of the cassava varieties grown by farmers. Let $E\left(T^{G}\right)$ be $p^{G}$ and denote $\kappa_{j}=Pr\left[T^{G}=1-j|T^{*}=j\right]$, for $j\in \left\{0,1\right\}$. Under this scenario, attempting to recover β via $T^{G}$ with the assumption that farmer perceptions are identical to the genetic test results leads to the following bias:^5^

(4) $$\begin{array}{ccc} \beta_{OLS}^{{}^{G}} & = & \frac{Cov\left(T^{G},X\right)}{Var\left(T^{G}\right)}=\frac{Cov\left(\beta T^{*}+{ɛ}^{*},T^{*}+\mu^{G}\right)}{Var\left(T^{G}\right)}\\ & = & \frac{\beta \left[Var\left(T^{*}\right)+Cov\left(T^{*},\mu^{G}\right)\right]}{Var\left(T^{G}\right)}\\ & = & \frac{\beta \left[p^{*}\left(1-p^{*}\right)\left(1-\kappa_{0}-\kappa_{1}\right)\right]}{p^{G}\left(1-p^{G}\right)}\\ & = & \frac{1}{p^{G}\left(1-p^{G}\right)}\left[\frac{\left(p^{G}-\kappa_{0}\right)\left(1-p^{G}-\kappa_{1}\right)}{1-\kappa_{0}-\kappa_{1}}\right]\beta \end{array}$$

Eq. (4) shows that $\left(\beta_{OLS}^{{}^{G}}\neq \beta \right)$ so long as $\kappa_{j}\neq 0$. Our characterization of the expected bias under misreporting in Eq. (3) and misperception in Eq. (4) underscores the importance of identifying the origins of misclassification. However, the origins of misclassification are unknown ex-ante and hence we cannot *a priori* determine whether $T^{G}$ or $T^{R}$ is the appropriate measure of $T^{*}$ in order to carry out inference on farmers’ input choices and input complementarity relationships. Therefore, drawing on the misclassification literature (e.g., Mahajan, 2006, Wilhelm, 2019), the next section introduces a non-parametric framework to carry out hypothesis tests for the presence of misclassification in $T^{R}$ or/and $T^{G}$.

### 2.2 Testing for misclassification

This section lays out a non-parametric framework for testing the presence of misclassification in $T^{R}$ or/and $T^{G}$. As discussed above, our central premise is based on the notion that when the underlying misclassification mechanism, misreporting or misperception, is unknown, $T^{R}$ and $T^{G}$ can only be considered as two surrogates of the true but unobserved variety perception ($T^{*}$). To fix ideas, we **maintain** the following non-differential misclassification assumption for input use throughout this paper.

Assumption 2.1 **Non-Differential Misclassification**

$E\left(X|T^{*},T^{R},T^{G}\right)=E\left(X|T^{*}\right)$

Assumption 2.1 is the conditional mean independence assumption and states that what matters to farmers’ input choices is their variety perceptions ($T^{*}$) instead of the two proxies, $T^{G}$ and $T^{R}$. Thus, it ensures that once $T^{*}$ is known, $T^{R}$ and $T^{G}$ are not informative about the conditional mean of X (i.e., $E\left(X|T^{*}\right)⊥\left(T^{G},T^{R}\right)|T^{*}$).^6^ Assumption 2.1 is standard in the measurement error literature (Mahajan, 2006, Wilhelm, 2019, Imai and Yamamoto, 2010). Hence, our hypothesis tests for the presence of misclassification in $T^{R}$ or/and $T^{G}$ relies on the validity of Assumption 2.1. The non-differential misclassification assumption of $T^{G}$ for input use is reasonable since the genetic-test results were not available to the farmers at the time of their input choices and genotyping error, if any, is likely to be random. Hence, the informational content of $T^{G}$ can only affect farmers’ input choices through its correlation with their variety perceptions (i.e., $T^{G}$ is expected to be a strong predictor of farmers’ variety perception formation ($T^{*}$) and conditional on $T^{*}$ it will not be informative about the conditional mean of X). Although the assumption of non-differential misclassification in $T^{R}$ is somewhat restrictive, it can be justified in our setting since all cassava farmers are subject to the same seed market shocks.

The exclusion restriction stated in Assumption 2.1 depends on the latent variety perception variable ($T^{*}$) and thus cannot be directly tested. However, as shown in Wilhelm (2019), testing the above exclusion restriction does not require solving for the distribution of the unobservable latent perceptions. Thus, we exploit our assumptions on the expected DGP of input use under misreporting and misperception to express Assumption 2.1 in terms of observables in our data (i.e., $T^{R}$ and $T^{G}$).

Assumption 2.2 **No Misreporting: $T^{*}=T^{R}$ w.r.t. Assumption 2.1**

Assumption 2.2 implies that farmer perceptions are equal to their reports. Under Assumption 2.1, Assumption 2.2, we can derive the following testable implication:

(5) $$E\left(X|T^{R},T^{G}\right)=E\left(X|T^{R}\right)$$

That is, under Assumption 2.1, the conditional mean of X in Eq. (5) does not depend on $T^{G}$ if and only if there is no misclassification in $T^{R}$ (Mahajan, 2006). The equality in (5) depends only on observables and can directly be tested without imposing any parametric assumptions about how the conditional mean of X depends on $T^{*}$ (Mahajan, 2006, Wilhelm, 2019, Lee and Wilhelm, 2019). It is, however, important to note that the equality in (5) is a joint test of Assumption 2.2, Assumption 2.1. Therefore, rejecting the equality in (5) implies that either one or both of Assumption 2.1, Assumption 2.2 do not hold. But failure to reject the equality does not imply that both assumptions are true, rather it implies that our data is consistent with the joint assumption.

Assumption 2.3 **No Misperception: $T^{*}=T^{G}$ w. r. t. Assumption 2.1**

Assumption 2.3 implies that farmer perceptions are equal to the genetic test results. Under Assumption 2.1, Assumption 2.3, we can derive the following testable implication:

(6) $$E\left(X|T^{G},T^{R}\right)=E\left(X|T^{G}\right)$$

Again, the testable implication in Eq. (6) is a joint test of Assumption 2.3, Assumption 2.1. However, under our maintained assumption that misclassification in the two proxies is non-differential for input use, the testable implications in Eqs. (5), (6) are informative on whether our data supports Assumption 2.2 or Assumption 2.3. Hence, under Assumption 2.1, if Eq. (5) holds but not Eq. (6), our data rejects Assumption 2.3 (i.e., $T^{*}=T^{G}$) and is consistent with Assumption 2.2 (i.e., $T^{*}=T^{R}$). On the other hand, if Eq. (6) holds but not (5), our data rejects Assumption 2.2 and is consistent with Assumption 2.3.

Alternatively, the above assumptions can also be tested by imposing linearity in the conditional expectations of the input use functions. As discussed above, the testable implication of Eq. (5) is equivalent to the restriction that conditional on $T^{R}$, $T^{G}$ is not informative about the conditional mean of X. Hence, imposing linearity, the testable implications of the above assumption can be reformulated as follows:

(7) $$X=\beta^{R}T^{R}+\beta^{G}T^{G}+{ɛ}^{RG}$$

Under Assumption 2.1, Assumption 2.2 implies that $\beta^{G}=0$ in Eq. (7). The hypothesis test in Eq. (7) relies on linearity of the conditional expectations of the input use functions and hence should only be considered as predictive/suggestive test.^7^ For instance, the test will be uninformative in the presence of misspecification (i.e., nonlinearities in the regression equations) since measurement error and nonlinearities can manifest themselves in similar ways (Wilhelm, 2019). In fact, in Eq. (7), testing for the presence of misclassification in $T^{R}$ is equivalent to testing for the exclusion restriction of $T^{G}$ (Mahajan, 2006, D’Haultfœuille et al., 2021).^8^

### 2.3 Point identification

The above non-parametric tests do not require solving for the distribution of the unobservable latent perceptions (i.e., estimation of the misclassification probabilities) and hence are very informative as a first step model specification test (Mahajan, 2006, Wilhelm, 2019). For instance, under Assumption 2.1, $E\left(X|T^{*}\right)$ can be point-identified via Eq. (3) if Assumption 2.2 holds or via Eq. (4) when Assumption 2.3 holds. However, if both assumptions are rejected, $E\left(X|T^{*}\right)$ cannot be point-identified either via Eq. (3) or Eq. (4).^9^ Following Mahajan (2006), this section describes the conditions for non-parametric identification of $E\left(X|T^{*}\right)$ using $T^{R}$ as a proxy for $T^{*}$ and $T^{G}$ as an instrumental-like variable (ILV) to deal with the potential measurement error in $T^{R}$. Hence, in addition to Assumption 2.1 above, we also maintain the following additional conditions.

Assumption 2.4 **Identification Assumptions**

(i) $T^{R}⊥T^{G}|T^{*}$
(ii) $P\left(T^{R}=1\left|T^{*}=0\right)+P\left(T^{R}=0\right|T^{*}=1\right)<1$
(iii) $P\left(T^{*}=1\left|T^{G}=1\right)\neq P\left(T^{*}=1\right|T^{G}=0\right)$

Assumption 2.4(i) states that conditional on $T^{*}$, $T^{G}$ and $T^{R}$ have independent sources of error. This assumption is expected to hold in our case because of the way the data are collected. In particular, $T^{R}$ originates from the survey (farmers) and $T^{G}$ from a genetic test, which is beyond farmers control as the genetic test results were not revealed to farmers at the time of their input choice (i.e., genotyping error, if any, originates from the laboratory analysis and hence is independent of the reporting errors by farmers in the survey conditional on their true variety perceptions). Hence, conditional on $T^{*}$, the error in $T^{G}$ has a very different origin from the error in $T^{R}$ as they originate from two independent sources. Assumption 2.4(ii) is the monotonicity condition and restricts the extent of misclassification in $T^{R}$ to ensure a positive correlation between $T^{R}$ and $T^{*}$. That is, the misclassification in $T^{R}$ is not so severe in the sense that $T^{R}$ is a better predictor of $T^{*}$ compared to $1-T^{R}$ (Mahajan, 2006, Hu, 2008, DiTraglia and García-Jimeno, 2019). Assumption 2.4(iii) is the instrumental variable relevance condition and ensures a strong correlation between $T^{G}$ and $T^{*}$ so that $T^{G}$ is informative about $T^{*}$.

Under Assumption 2.1, Assumption 2.4, misreporting rates (i.e., $\alpha_{0}$ and $\alpha_{1}$) can be point-identified as a function of the observable moments of ($T^{R}$, X, $XT^{R}$) in our data.^10^ To see this, let us define the relationship between $T^{*}$ and $T^{R}$ in relation to $T^{G}=j$ where $j\in \left\{1,2\right\}$ since $T^{G}$ is binary as follows: Denote $p_{j}^{*}=P\left(T^{*}=1|T^{G}=j\right)$; and $p_{j}=P\left(T^{R}=1|T^{G}=j\right)$. Using the law of total probability, the relationship between $p_{j}^{*}$ and $p_{j}$ is given as:

(8) $$p_{j}^{*}=\frac{p_{j}-\alpha_{0}}{1-\alpha_{0}-\alpha_{1}};\;1-p_{j}^{*}=\frac{1-p_{j}-\alpha_{1}}{1-\alpha_{0}-\alpha_{1}}$$

Hence, if $\alpha_{0}$ and $\alpha_{1}$ are known, $p_{j}^{*}$ will be identified via Eq. (8) and $E\left(X|T^{*}\right)$ via Eq. (3) since $p_{j}$ is observable in our data. Thus, we can estimate $E\left(X|T^{*}\right)$ consistently using $T^{G}$ as ILV to deal with the misclassification in $T^{R}$ using a Generalized Method of Moments (GMM) estimator.

### 2.4 Implications for inference on productivity

In this section, we aim to show the inferential implications of misclassification focusing on the reduced form biophysical relationship between improved genetics and yield (Y) (i.e., cassava output per hectare). However, the non-differential misclassification assumption of the two proxies for input use (i.e., Assumption 2.1) is unlikely to hold for yield since $T^{G}$ is expected to directly affect cassava yields over and above its indirect effect via $T^{*}$ and X (i.e., $E\left(Y|T^{*},T^{G},T^{R},X\right)\neq E\left(Y|T^{*},X\right)$ since improved varieties generate higher yields even after conditioning on $T^{*}$ and X). Hence, with the additional assumption that $T^{G}$ measures the true genetic quality of cassava varieties grown by farmers without error, we choose to specify the reduced form biophysical relationship using $T^{G}$ instead of the latent perception variable ($T^{*}$). Since $T^{G}$ is observed in our data, estimating the reduced form biophysical relationship between $T^{G}$ and Y is rather straightforward in the absence of variety misperception (i.e., $\left(T^{*}=T^{G}\right)\neq T^{R}$ w.r.t. Assumption 2.1). Under this scenario, the relevant unconditional non-differential measurement error assumption for yield becomes:

Assumption 2.5 $E\left(Y\left|T^{*},T^{G},T^{R}\right)=E\left(Y\right|T^{*},T^{G}\right)$

Assumption 2.5 states that farmer reports are not informative about yields once we know the genetic test results as well as farmers perception at input choice time. When $T^{*}=T^{G}$, the testable implication of Assumption 2.5 states that conditional on $T^{G}$, farmer reports provide no additional information about the conditional mean of Y.^11^ Assuming a linear relationship, the reduced form biophysical relationship between the true genetic quality of the cassava varieties grown by farmers ($T^{G}$) and Y can be specified using the following production function:

(9) $$Y=\gamma^{G}T^{G}+\omega^{G}$$

To demonstrate the expected bias due to variety misreporting, the error term, $\omega^{G}$, is assumed to be mean zero and uncorrelated with $T^{G}$.^12^ As before, let us assume that the researcher has access to farmer reports ($T^{R}$) only, which is a misclassified version of $T^{G}$. Recalling $T^{R}=\left(T^{*}+\mu^{R}\right)\equiv \left(T^{G}+\mu^{R}\right)$ in the presence of only variety misreporting, the production function estimated by the researcher is given by: $Y=\gamma^{G}T^{R}+\left(\omega^{G}-\gamma^{G}\mu^{R}\right)$. Hence, estimating Eq. (9) using $T^{R}$ instead of $T^{G}$ leads to the following bias:

(10) $${\overset{ˆ}{\gamma }}^{R}=\frac{Cov\left(Y,T^{R}\right)}{Var\left(T^{R}\right)}=\frac{Cov\left(\gamma^{G}T^{G}+\omega^{G},T^{G}+\mu^{R}\right)}{Var\left(T^{R}\right)}\neq \gamma^{G}$$

Since $Cov\left(T^{G},\mu^{R}\right)<0$, due to the non-classical nature of variety misreporting, it follows that ${\overset{ˆ}{\gamma }}^{R}<\gamma^{G}$. In this case, estimating Eq. (10) using $T^{G}$ in stead of $T^{R}$ would completely overcome the bias in ${\overset{ˆ}{\gamma }}^{R}$.

If the testable implication of Assumption 2.5 is rejected (i.e., conditional on $T^{G}$, $T^{R}$ provides relevant information about the conditional mean of Y), it must be the case that either $T^{*}\neq T^{G}$ w.r.t. Assumption 2.1 or/and Assumption 2.5 do not hold for yield. In fact, in the presence of misperception, since input use decisions are made based on perceived ($T^{*}$) instead of true genetic quality ($T^{G}$), misperception will introduce an endogeneity problem in Eq. (9) (i.e., $\gamma^{G}$, the OLS estimator of Eq. (11) could be biased since the assumption that $Cov\left(T^{G},\omega^{G}\right)=0$ will not hold). Hence, we reformulated our non-differential measurement error assumption for yield as follows:

Assumption 2.6 $E\left(Y\left|T^{*},T^{G},T^{R},X\right)=E\left(Y\right|T^{*},T^{G},X\right)$

Assumption 2.6 invokes the non-differential measurement error assumption conditional on farmers perception at input choice time ($T^{*}$), ex-post input choices (X) and the true genetic quality of the cassava varieties grown by farmers ($T^{G}$). When $T^{*}=T^{G}$, the testable implication of Assumption 2.6 states that farmer variety reports are not informative about the conditional mean of Y once we know the genetic test results and farmers’ ex-post input choices (X). Given the above non-differential variety misperception assumption for yield, the reduced form biophysical relationship between $T^{G}$ and Y can be re-formulated as follows:

(11) $$Y=\gamma^{G}T^{G}+\delta X+\omega^{gx}$$

Assuming $\omega^{gx}$ is mean zero and uncorrelated with both X and $T^{G}$, the OLS estimator of Eq. (11) will be unbiased. As shown in Eq. (10) above, when estimating Eq. (11) using $T^{R}$ instead of $T^{G}$, we implicitly assume the following regression specification: $Y=\gamma^{G}T^{R}+\delta X+\left(\omega^{gx}-\gamma^{G}\mu^{R}\right)$. In this case, the expected bias in ${\overset{ˆ}{\gamma }}^{R}$ depends not only on the non-classical nature of variety misreporting (i.e., $Cov\left(T^{G},\mu^{R}\right)<0$) but also on whether $Cov\left(X,\mu^{R}\right)$ is zero or not. In particular, even if both $T^{G}$ and X are expected to be exogenous in Eq. (11), the correlation between X and $\mu^{R}$ cannot be assumed to be zero as long as $\left(T^{*}\equiv T^{G}\right)$ is strongly correlated with X (Nguimkeu et al., 2021).^13^

Since true genetic quality ($T^{G}$) is assumed to be measured without error, rejecting the equality implied by the observable implication of Assumption 2.6 would imply that either Eq. (11) does not include all the relevant input choices (i.e., all the relevant inputs were not included in X) or/and measurement error is differential for yield in the sense that farmer perceptions affect yields not only through input choices but also via other channels. For the sake of exposition, let us assume that farmer perceptions affect yields only through input choices. In particular, imposing this additional assumption, Assumption 2.6 can be re-formulated as follows:

Assumption 2.7 $E\left(Y\left|T^{*},T^{G},T^{R},X\right)=E\left(Y\right|T^{G},X\right)$

Under Assumption 2.7, conditional on X and $T^{G}$, farmer variety perceptions or reports will not be informative about the conditional mean of Y. In fact, the assumption that farmer perceptions only affect yields through input choices is equivalent to the restriction that conditional on X, $T^{*}=T^{G}$ w.r.t. Assumption 2.5. As discussed above, in the presence of misperception, input adjustments that were not included in X will remain in the error term of Eq. (11) (i.e., our assumption that $\omega^{gx}$ is mean zero and uncorrelated with both X and $T^{G}$ will not hold). However, imposing Assumption 2.7, we can probe the relevance of misperception by conditioning our regressions on $T^{G}$, X and $T^{R}$ in spirit of control function approach. That is, since both $T^{G}$ and $T^{R}$ measure the same concept (i.e., the genetic quality of the cassava varieties grown by farmers) and given that $T^{G}$ is assumed to measure genetic quality without error, we are assuming that conditional on $T^{G}$ and X, $T^{R}$ is expected to capture misperception induced input choices that were not included in X. Under this scenario, the appropriate regression specification for yield becomes:

(12) $$Y=\gamma^{G}\left(m\right)T^{G}+\Delta X+\Phi T^{R}+\omega^{m}$$

Since $\omega^{m}$ is mean zero and uncorrelated with X and $T^{G}$ conditional on $T^{R}$ by Assumption 2.7, the OLS estimator of Eq. (12) will be unbiased.^14^ In fact, under Assumption 2.7, if all the relevant input choices are captured by X as assumed in Eq. (11), the coefficient associated with $T^{R}$ is expected to be statistically insignificant (i.e., Φ=0 or conditional on X and $T^{G}$, $T^{R}$ is not relevant for Y).^15^

## 3 Data and descriptive statistics

For examining the implications of variety misclassification, we use data from the Cassava Monitoring Survey (CMS) of Nigeria.^16^ The CMS was conducted in 2016 with the aim of measuring the adoption rate of improved cassava cultivars in Nigeria. To do so, data were collected from 16 states, which together account for more than 80 percent of total cassava production of the country. To collect nationally representative data, a multistage stratified sampling design was employed. First, the 16 states were grouped into four geopolitical regions. Then, 125 enumeration areas (EAs) were selected from each region using probability proportional to size (PPS) sampling approach. From each EA, 5 cassava growing households were then randomly selected for interview. During the post-planting survey, detail information about the type of cassava varieties grown by farmers were collected using two different approaches. The first is by asking farmers to report the type of cassava variety they grow, specifically whether the cassava variety they grow is improved or not. This corresponds to the standard variety data collection approach often employed in household surveys.^17^ Second, leaf samples from all identified cassava plots of farmers were also collected to accurately identify the improvement status of the cassava varieties grown by farmers through DNA-fingerprinting. Since the DNA-fingerprinting approach is independent of environmental conditions or plant growth stage, the improvement status of the cassava varieties grown by an individual farmer can be identified accurately (Rabbi et al., 2015).^18^ We then match farmer reports with the genetic-test results to explore the extent of mismatch in the two variety identification methods.

Table 1 presents summary statistics associated with variety identification, input use and other household and plot characteristics.^19^ In our analysis, farmers’ report is coded as a binary indicator using farmers’ response on the improvement status of the cassava varieties they grow. That is, for each identified cassava plot, farmers’ report takes a value of one if the farmer identifies the cassava variety as improved and zero otherwise. Similarly, for each identified cassava plot, the genetic test result is coded as one if the cassava variety is confirmed to be improved and zero otherwise. According to farmer reports, about 56% of the plots are planted with improved cassava varieties but the genetic test results show that 70% of the plots are planted with improved cassava varieties. From the matched plot level variety information, we find mismatches between farmer reports and the genetic test results in about 36% of the plots in the full sample (i.e., while plots with $\left(T^{R}=0;T^{G}=1\right)$ and $\left(T^{R}=1;T^{G}=0\right)$ cases account about 25% and 11% of the full sample, plots with $\left(T^{R}=0;T^{G}=0\right)$ and $\left(T^{R}=1;T^{G}=1\right)$ cases account about 19% and 45% of the full sample, respectively).^20^ As discussed in Section 2, since the underlying misclassification mechanism is unknown ex-ante, the above reported mismatches between farmer reports and genetic-test results suggest the presence of either variety misperception or misreporting in our data.^21^

**Table 1:** Descriptive statistics.

	Mean	Standard deviation
Improved variety (T${}^{R}$)	0.56	0.49
Improved variety (T${}^{G}$)	0.70	0.46
Fertilizer use (1 = Yes; 0 = No)	0.26	0.44
Fertilizer (kg/ha)	22.9	50.4
Herbicide use (1 = Yes; 0 = No)	0.47	0.50
Herbicide (lit/ha)	6.18	12.35
Labor ( in person-days)	55.25	88.6
Yield (t/ha)	14.81	9.64
Household size (# members)	4.6	2.4
Age of household head	51.7	13.7
Education of household head (Years of schooling)	8.7	4.9
Gender of household head (1 = Male, 0 = Female)	0.9	0.3
Access to extension (1 = Yes; 0 = No)	0.36	0.48
Membership in cassava growers’ association (1 = Yes; 0 = No)	0.21	0.41
Plot manager (1 = Men, 0 = Jointly/Women)	0.37	0.48
Plot soil fertility status (1 = Good, 0 = Medium/Poor)	0.74	0.44
Plot distance from residence (kilometers)	2.02	2.1

No. observations	3933	

From each cassava plot, we also elicited production and input use information. For instance, fertilizer and herbicide inputs were applied in about 26% and 47% of the plots, respectively. To uncover farmers’ observed input use behavior, we report the means of fertilizer and herbicide input use conditional on farmer reports and genetic test results in Table 2. Results suggest a positive correlation between adoption of improved cassava varieties and input use decisions. However, this correlation is much stronger conditional on farmer reports compared to the genetic test results. In Table 2, we also report the mean of cassava yield given farmer reports and genetic-test results. Unlike input use patterns, cassava yields are much higher conditional on $T^{G}$ compared to $T^{R}$. The above empirical pieces of evidence suggest that while farmers’ variety report is strongly correlated with their input use behavior, yields seem to be more aligned with the true genetic attributes of the cassava varieties grown by farmers. We will formally test these empirical patterns using both parametric and non-parametric methods in Section 5.

**Table 2:** Conditional means of yield, fertilizer and herbicide inputs.

Variable	(1)	(2)	(3)	(4)	(5)	T-test difference	
	$T^{R}=0$	$T^{R}=1$	$T^{G}=0$	$T^{G}=1$	Total		
	Mean/SE	Mean/SE	Mean/SE	Mean/SE	Mean/SE	(2)-(1)	(4)-(3)
Fertilizer use	0.199 (0.010)	0.312 (0.010)	0.229 (0.012)	0.276 (0.009)	0.262 (0.007)	0.113***	0.047***
Herbicide use	0.259 (0.010)	0.640 (0.010)	0.370 (0.014)	0.515 (0.010)	0.472 (0.008)	0.381***	0.145***
Yield (t/ha)	13.485 (0.211)	15.869 (0.217)	11.844 (0.236)	16.08 (0.19)	14.812 (0.154)	2.384***	4.076***
No. observations	1744	2189	1175	2758	3933		

*Notes*:The value displayed for t-tests are the differences in the means across the groups. ***, **, and * indicate significance at the 1, 5, and 10 percent critical level.

## 4 Estimation

We now describe the empirical strategy we employed to estimate the quantities of interest. We first start by carrying out the non-parametric tests described in Section 2.2 for each input, both at the intensive and extensive margin of input use. Under our maintained assumption that misclassification in the two proxies is non-differential for input use, we test the null of no misclassification in $T^{R}$ (i.e., the hypothesis that farmer perceptions are equal to their reports as stated in Assumption 2.2) by running a non-parametric regression of $X_{ip}$ on $T_{ip}^{R}$ and $T_{ip}^{G}$ as follows:

(13) $$H_{0}^{T^{R}=T^{*}}:E\left(X_{ip}\left|T_{ip}^{R},T_{ip}^{G}\right)=E\left(X_{ip}\right|T_{ip}^{R}\right)$$

where $X_{ip}$ stands for observed input use by household i at plot p. That is, under Assumption 2.1, the conditional mean of X does not depend on $T^{G}$ if and only if there is no misclassification in $T^{R}$. Hence, $H_{0}^{T^{R}=T^{*}}$ can be considered as a test of misreporting in the sense that poor measurement derives misclassification in our data.

Similarly, we test the null of no misclassification in $T^{G}$ (i.e., the hypothesis that farmer perceptions are equal to the genetic test results as stated in Assumption 2.3) as follows:

(14) $$H_{0}^{T^{G}=T^{*}}:E\left(X_{ip}\left|T_{ip}^{G},T_{ip}^{R}\right)=E\left(X_{ip}\right|T_{ip}^{G}\right)$$

Under Assumption 2.1 the conditional mean of X does not depend on $T^{R}$ if and only if there is no misclassification in $T^{G}$. Hence, $H_{0}^{T^{G}=T^{*}}$ is a test of no misperception in the sense that market frictions such as asymmetric quality information derives misclassification in our data (Wilhelm, 2019, Lee and Wilhelm, 2019). That is, in the absence of market frictions, farmers’ input allocation decision is expected to be based on the true genetic quality of the crop varieties they grow (i.e., based on $T^{G}=T^{*}$). We carry out the above hypothesis tests for the null of no misclassification in the two proxies using the Cramer–von Mises (CvM) and Kolmogorov–Smirnov (KS) test statistics. Under Assumption 2.1, if Eq. (13) holds but not Eq. (14), misperception is considered to be the main source of variety misclassification in our data. On the other hand, if Eq. (14) holds but not (13), misreporting is considered to be the main source of variety misclassification in our data.

Next, we carry out hypothesis tests for the presence of misclassification under parametric model specification and examine the inferential and behavioral implications of misclassification. To examine the inferential implication of misclassification, we follow our analytical framework and estimate input complementarity relationships based on the OLS estimator from a regression of X on a constant and $T^{R}$ as well as X on $T^{G}$ and a constant. As discussed in Section 2.1, in terms of inference on input complementarity relationships, $E\left(X|T^{*}\right)$ can be point-identified via Eq. (3) if Assumption 2.2 holds or via Eq. (4) when Assumption 2.3 holds. Thus, following our analytical framework, we estimate the following adoption propensity equations:^22^

(15a) $$X_{ip}=\theta^{RG}+\beta^{R}T_{ip}^{R}+\beta^{G}T_{ip}^{G}+{ɛ}_{ip}^{RG}$$

(15b) $$X_{ip}=\theta^{FE}+\beta^{R\tau }T_{ip}^{R}+\beta^{G\tau }T_{ip}^{G}+\tau_{i}+{ɛ}_{ip}^{FE}$$

where all terms are as defined above and $\tau_{i}$ stands for household fixed effects.^23^ Imposing linearity in the input equations, Eqs. (15a), (15b) tests the restriction that conditional on $T^{R}$, $T^{G}$ is not informative about the conditional mean of X. Under our maintained assumption that misclassification in $T^{R}$ and $T^{G}$ is non-differential for input use, $\beta^{G}=0$ and $\beta^{R}\neq 0$ in these regression specifications imply that $T^{R}$ is a more accurate measure of $T^{*}$ compared to $T^{G}$. In this case, the OLS estimator from a regression of X on $T^{R}$ (and a constant) is expected to be unbiased (i.e., $E\left(X|T^{R}\right)\equiv E\left(X|T^{*}\right)$). On the other hand, $\beta^{R}=0$ and $\beta^{G}\neq 0$ implies that $T^{G}$ is a more accurate measure of $T^{*}$ compared to $T^{R}$. In this case, the OLS estimator from a regression of X on $T^{G}$ (and a constant) is expected to be unbiased (i.e., $E\left(X|T^{G}\right)\equiv E\left(X|T^{*}\right)$).

Next, we explore the behavioral implications of misclassification, focusing on farmers’ observed input use and intensification choices. To facilitate comparisons, we introduce a discrete variable, $V_{ipc}$, which assumes the following values depending on the value of $T^{R}$ and $T^{G}$.

(16) $$V_{ipc}=\left\{\begin{array}{cc} 0,\; & \left(T_{ip}^{G};T_{ip}^{R}\right)=1\\ 1,\; & T_{ip}^{G}=0;T_{ip}^{R}=1\\ 2,\; & T_{ip}^{G}=1;T_{ip}^{R}=0\\ 3,\; & \left(T_{ip}^{G};T_{ip}^{R}\right)=0 \end{array}\right)$$

Using the above relationship, we then estimate the following regression specification:

(17) $$X_{ip}=\theta_{c}+\phi_{c}V_{c}+\lambda Z_{ip}+{ɛ}_{ip}^{v}$$

where all terms are as defined above and $Z_{ip}$ are other determinants of input use. Unlike the more simplified analytical frameworks, our empirical specification in Eq. (17) is slightly saturated and hence accounts for additional household and plot level characteristics.^24^ Assuming a positive relationship between adoption of improved cassava varieties and input use and letting $V_{3}$ as the base category, we expect a positive and statistically significant coefficient on $\phi_{0}$ and $\phi_{1}$ but an insignificant coefficient on $\phi_{2}$ in the presence of misperception. Similarly, letting $V_{0}$ as the base category, $\phi_{1}$ is expected to be statistically insignificant while $\phi_{2}$ and $\phi_{3}$ are expected to be negative and significant in the presence of variety misperception. In terms of inference, the OLS estimator of $\phi_{0}$ in Eq. (17) can also be used to establish the lower bounds of $E\left(X|T^{*}\right)$ (Black et al., 2000). In particular, under our maintained assumptions and assuming $E\left(X|T^{*}\right)>0$ the following condition is expected to hold in our data: $\left(E\left(X|T^{R}\right)\equiv \beta_{OLS}^{R}\right)\leq \phi_{0}\leq \left(E\left(X|T^{*}\right)\equiv \beta \right)$.

Once we examined the behavioral and inferential implications of misclassification on input use and intensification choices, we extend and test the inferential implications of misclassification on productivity. We do so, for demonstrating two objectives: (i) we aim to show the implication of misclassification to inference related to yield, (ii) we also aim to explore whether input crowding-in(out) driven by misperception are yield-enhancing or not. To demonstrate this, we estimate the following linear regression specifications:

(18a) $$Y_{ip}=\Delta^{R}+\gamma {}^{R}T_{ip}^{R}+\delta^{r}X_{ip}+\phi^{r}Z_{ip}+\tau_{i}+\eta_{ip}^{R}$$

(18b) $$Y_{ip}=\Delta^{G}+\gamma^{G}T_{ip}^{G}+\delta^{g}X_{ip}+\phi^{g}Z_{ip}+\tau_{i}+\eta_{ip}^{G}$$

(18c) $$Y_{ip}=\Delta^{GR}+\gamma^{G}T_{ip}^{G}+\delta^{gr}X_{ip}+\phi^{gr}Z_{ip}+\zeta T_{ip}^{R}+\tau_{i}+\eta_{ip}^{GR}$$

where $Y_{ip}$ stands for log-transformed cassava yield for each plot. As discussed in Section 2.4, Eq. (18a) generates biased estimates due to the non-classical nature of variety misclassification. Eq. (18b) is estimated conditional on $T^{G}$ and X (i.e., to account for input adjustments). For the purpose of hypothesis testing, we also estimate Eq. (18c) (i.e., as explicated in Eq. (12), with the assumption that misperception affects yields only through input choices, conditional on $T^{G}$ and X, ζ is expected to be insignificant.) Finally, we examine the inferential implication of misperception and associated input adjustments on the marginal yield response of inputs using the following empirical specification:

(19) $$Y_{ip}=\Delta^{ipc}+\varphi_{c}V_{c}+\Phi X_{ip}+\rho_{c}\left(V_{c}*X_{ip}\right)+\Psi Z_{ip}+\tau_{i}+\omega_{ip}$$

where all terms are as defined above. To quantify the implications of input allocation driven by misperception, we interact $V_{c}$ with $X_{ip}$. These interaction terms can inform whether mis(perception) driven allocation of inputs such as fertilizer is yield-enhancing or not. For instance, if misperception induced crowding-in of fertilizer has no discernible influence on yield $\left(\rho_{c}=0\right)$, then the allocation of fertilizer can be argued to be inefficient.

## 5 Results

### 5.1 Non-parametric and parametric test results

In this section, we present the results of our formal non-parametric and parametric tests carried out to identify the presence of misclassification in $T^{R}$ and $T^{G}$. As discussed in Section 2.2, these tests gauge whether farmer perceptions are equal to their reports (i.e., $T^{*}=T^{R}$) or the genetic test results (i.e., $T^{*}=T^{G}$). For each input use, both at the extensive and intensive margins, the results of the non-parametric tests are reported in Panel A of Table 3. For each input, the first two column report the results of the unconditional and conditional version of our tests, respectively. In the conditional version of our test, the non-differential misclassification assumption of the two proxies for input use is expected to hold conditional on $T^{*}$ and other additional controls (z) (i.e., $E\left[X\left|T^{*},T^{R},T^{G},z\right]=E\left[X\right|T^{*},z\right]$. We use the p-value of both the Cramer–von Mises (CvM) and Kolmogorov–Smirnov (KS) test statistics to compare the equality of the input use expectations (i.e., null hypothesis of no misclassification in the two proxies).

In the first two rows of Panel A, we test whether farmer perceptions are equal to their reports (i.e., $T^{*}=T^{R}$). In this case, conditional on $T^{R}$, $T^{G}$ is expected to be uninformative about the conditional mean of X. In the unconditional version of our tests, except for herbicide input use at the extensive margin, both the CvM and KS versions of our tests consistently fail to reject the null of no misclassification in $T^{R}$ (i.e., $E\left[X\left|T^{R},T^{G}\right]=E\left[X\right|T^{R}\right]$). In the conditional version of our tests, we consistently fail to reject the null of no misclassification in $T^{R}$ (i.e., $E\left[X\left|T^{R},T^{G},z\right]=E\left[X\right|T^{R},z\right]$. Following a similar logic, we also test whether farmer perceptions are equal to the genetic test results in the third and fourth rows of Panel A. In this case, conditional on $T^{G}$, $T^{R}$ is expected to be uninformative about the conditional mean of X. Both the conditional and unconditional version of our tests consistently reject the null of no misclassification in $T^{G}$ (i.e., $E\left[X\left|T^{G},T^{R}\right]\neq E\left[X\right|T^{G}\right]$). Hence, under Assumption 2.1, our data rejects the assumption of no misclassification in $T^{G}$ and is consistent with the assumption of no misclassification in $T^{R}$.

Imposing linearity, we also report additional predictive parametric tests by regressing X on both $T^{R}$ and $T^{G}$ (and a constant) in Panel B and C of Table 3. Under our maintained assumptions, the hypothesis that $T^{R}=T^{*}$ holds if $\beta^{G}=0$ and ${\overset{ˆ}{\beta }}^{R}\neq 0$. Test results reported in Panel B and C of Table 3 show that, $\beta^{G}=0$ and ${\overset{ˆ}{\beta }}^{R}\neq 0$ holds in our data. In addition, the p-values of the Naive Wald statistics reported at the bottom of Panel B shows that the exclusion restriction of $T^{G}$ is satisfied in our data.^25^ The p-values of the Lochner–Moretti (LM) statistics, which is robust to misspecification (i.e., nonlinearities in the regression specifications), further confirms that the exclusion restriction of $T^{G}$ is satisfied in our data (Lochner and Moretti, 2015). Thus, consistent with our non-parametric tests, the results reported in Panel B and C of Table 3 support the hypothesis that farmer perceptions are equal to their reports but not with the genetic-test results.

However, since $T^{G}$ is expected to measure the true genetic quality of the cassava varieties grown by farmers more accurately compared to $T^{R}$, the above test results imply that the main source/origin of variety misclassification in our data is misperception instead of misreporting. Note that, since our non-parametric tests rely on the weaker conditional mean independence assumption (i.e., Assumption 2.1), instead of the absence of misclassification in $T^{R}$, the above test results imply misclassification in $T^{R}$, if any, will not distort the conditional mean of X (Wilhelm, 2019).^26^ That is, for randomly drawn cassava farmers, the following condition hold in our data: $E\left(X|T^{*}\right)=E\left(X|T^{R}\right)$ but $E\left(X|T^{*}\right)\neq E\left(X|T^{G}\right)$. Our results slightly differ from those documented by Abay et al. (2021), who reported that measurement error in plot size represent a mixture of misreporting and misperceptions.^27^ However, our results are intuitive in the sense that farmers are more likely to be prone to variety misperception due to the credence nature of crop variety traits.

**Table 3:** Test results for the presence of misclassification.

Panel A: Non-parametric test results								

$T^{*}=T^{R}$ w.r.t. $E\left[X|T^{*},T^{R},T^{G}\right]=E\left[X|T^{*}\right]$								

	Fertilizer				Herbicide			
	dummy		kg/ha		dummy		lit/ha	
p(CvM < CvM${}^{*}$)	0.222	0.328	0.746	0.438	0.016	0.332	0.154	0.884
p(KS < KS${}^{*}$)	0.174	0.558	0.622	0.638	0.006	0.208	0.108	0.836

$T^{*}=T^{G}$ w.r.t. $E\left[X|T^{*},T^{G},T^{R}\right]=E\left[X|T^{*}\right]$								

p(CvM < CvM${}^{*}$)	0.000	0.000	0.000	0.000	0.000	0.000	0.000	0.000
p(KS < KS${}^{*}$)	0.000	0.000	0.000	0.000	0.000	0.000	0.000	0.000

(#) of bootstrap replications	500	500	500	500	500	500	500	500

Panel B: Parametric test results								

${\overset{ˆ}{\beta }}^{R}T^{R}$	0.109***	0.107***	0.667***	0.661***	0.372***	0.320***	0.783***	0.674***
	(0.023)	(0.023)	(0.115)	(0.115)	(0.028)	(0.030)	(0.08)	(0.088)
${\overset{ˆ}{\beta }}^{G}T^{G}$	0.017	0.017	0.043	0.042	0.044	0.023	0.071	0.007
	(0.024)	(0.024)	(0.117)	(0.116)	(0.029)	(0.029)	(0.086)	(0.088)

Naive Wald	0.15	0.153	0.468	0.473	0.001	0.131	0.097	0.892
LM-Wald	0.264	0.266	0.581	0.585	0.011	0.188	0.192	0.906

Panel C: Parametric test results with household fixed effects								

${\overset{ˆ}{\beta }}^{R}T^{R}$	0.141***	0.141***	0.860***	0.860***	0.294***	0.294***	0.526***	0.526***
	(0.039)	(0.04)	(0.199)	(0.199)	(0.048)	(0.048)	(0.160)	(0.160)
${\overset{ˆ}{\beta }}^{G}T^{G}$	−0.006	−0.006	−0.071	−0.07	0.009	0.009	−0.058	−0.058
	(0.033)	(0.033)	(0.148)	(0.148)	(0.031	(0.031)	(0.105)	(0.105)

Other controls	No	Yes	No	Yes	No	Yes	No	Yes
No. observation	3933	3933	3933	3933	3933	3933	3933	3933

*Note.* For parametric tests, standard errors clustered at enumeration area level are reported in parentheses. * p<0.10, ** p<0.05, *** p<0.01. For input use at the intensive margin, we employ inverse hyperbolic sine (IHS) transformation to keep observations with zero input use. The conditioning variable in the fertilizer input equation is distance to the nearest fertilizer dealer. In the herbicide input equation, the conditioning variables are self-reported incidence of cassava pest and herbicide price. The FE estimates for the unconditional and conditional version of our tests are the same since the conditioning variables are plot invariant.

### 5.2 Point identification results

In this section, we directly explore the inferential implications of misclassification focusing on the estimation of input complementarity relationships. As discussed in Section 2.3, the results of the above non-parametric tests are informative about the appropriate estimation strategies since the input complementarity relationships estimated using $T^{R}$ and $T^{G}$ are expected to be unbiased in the absence of misreporting and misperception, respectively. In our case, since both the parametric and non-parametric tests consistently fail to reject the hypothesis that farmer perceptions are equal to their reports, we expect the difference between $E\left(X|T^{*}\right)$ and $E\left(X|T^{R}\right)$ to be statistically insignificant. To confirm this, we report the GMM point-estimates of $E\left(X|T^{*}\right)$ as well as the naive OLS and IV estimates that ignores misclassification in Table 4. In particular, for each input, both at the extensive and intensive margins, we report the GMM point-estimates of $E\left(X|T^{*}\right)$ in the first row. We then report OLS estimates of $E\left(X|T^{R}\right)$ and $E\left(X|T^{G}\right)$ using $T^{R}$ and $T^{G}$ as a proxy for $T^{*}$ (i.e., by regressing X on $T^{R}$ (and a constant) and X on $T^{G}$ (and a constant), respectively) in the second and third rows, respectively. The fourth row reports the results of our parameter equality tests (i.e., $E\left(X|T^{R}\right)=E\left(X|T^{G}\right)$).

Consistent with our non-parametric test results, estimates reported in Table 4 suggest that the GMM point-estimates of $E\left(X|T^{*}\right)$ are almost identical with the OLS estimates that uses $T^{R}$ as a proxy for $T^{*}$ (i.e., $E\left(X|T^{*}\right)\equiv E\left(X|T^{R}\right)$). For instance, for fertilizer use (kg/ha), estimates of $E\left(X|T^{R}\right)$ and $E\left(X|T^{*}\right)$ are 0.677 and 0.742, respectively, suggesting the bias in $E\left(X|T^{R}\right)$ is only about 9 percent. Note that, the difference between the two conditional means is expected to be statistically insignificant (i.e., $E\left(X|T^{*}\right)=E\left(X|T^{R}\right)$ as confirmed in our non-parametric test results above). On the other hand, the OLS estimator that uses $T^{G}$ as a proxy for $T^{*}$ (i.e., $E\left(X|T^{G}\right)$) yields estimates of $E\left(X|T^{*}\right)$ that are biased towards zero by up to 70 percent. Under our maintained assumptions, the difference between the GMM point-estimates of $E\left(X|T^{*}\right)$ and $E\left(X|T^{G}\right)$ is expected to be caused by measurement error in $T^{G}$ (i.e., misperception). In fact, since our GMM estimation approach point identifies the misclassification rates in $T^{R}$ and $T^{G}$, we can also get a sense of how different the farmer reports and genetic test results are from the true, but unobserved, perception ($T^{*}$). Inspecting the estimated misclassification rates reported in Table 4, we can see that the estimated misclassification rate in $T^{R}$ is small (albeit non-zero) and mostly statistically insignificant but the estimated misclassification rate in $T^{G}$ is very high and statistically significant.^28^ In particular, while the maximum misreporting rate derived from our GMM estimation approach is about 15% (i.e., measurement error in $T^{R}$ denoted by: $\alpha_{t}=Pr\left[T^{R}=1-t|T^{*}=t\right]$, for $t\in \left\{0,1\right\}$), the corresponding misperception rate in our data is about 75%(i.e., measurement error in $T^{G}$ denoted by: $\kappa_{j}=Pr\left[T^{G}=1-j|T^{*}=j\right]$, for $j\in \left\{0,1\right\}$)).

Given the estimates reported in Table 4 (i.e., $E\left(X|T^{*}\right)\equiv E\left(X|T^{R}\right)$), the inferential implications of misperception can thus be inferred by probing the equality of $E\left(X|T^{R}\right)$ and $E\left(X|T^{G}\right)$. As expected, the parameter equality test reported in the fourth row of Table 4 (i.e., $E\left(X|T^{R}\right)-E\left(X|T^{G}\right)$) detects statistically significant difference in the two conditional means. In fact, we reject the null of parameter equality for all inputs, both at the intensive and extensive margins. This comparison reveals that the estimated input complementarity relationship is stronger when using farmer reports instead of genetic-test results as a proxy to farmers’ unobserved but true variety perceptions. Therefore, failure to identify the origins of misclassification could undermine inference related to input complementarity relationships, implying that treating misperception as survey misreporting and vice versa can lead to potentially wrong policy prescriptions. For instance, if we wrongly assume misreporting instead of misperception as the source of variety misclassification (i.e., this amounts imposing the assumption that $T^{G}$ is a more reliable and accurate measure of both the unobserved but true variety perceptions of farmers ($T^{*}$) and the genetic quality of the cassava varieties grown by farmers compared to $T^{R}$), inference on input complementary relationships relies on $T^{G}$ instead of $T^{R}$. However, the parameter equality tests reported in the fourth row of Table 4 suggest that $E\left(X|T^{R}\right)$ is almost threefold of $E\left(X|T^{G}\right)$.

To provide additional perspectives on the inferential implications of misperception, we also report the naive IV estimates that ignores misclassification from a regression of X on $T^{R}$ using $T^{G}$ as instrument (i.e., $\beta_{IV}^{R}$) and a regression of X on $T^{G}$ using $T^{R}$ as instrument (i.e., $\beta_{IV}^{G}$) in the seventh and eighth rows of Table 4, respectively. In the final row, we also report OLS estimates from a restricted sample by discarding observations with conflicting $T^{G}$ and $T^{R}$ values as if these observations were missing at random. Under our maintained assumptions, we expect the naive IV estimates to be larger in magnitude relative to their OLS counterparts and the GMM point-estimates of $E\left(X|T^{*}\right)$ since non-differential measurement error in our setting is expected to inflate the IV estimates (Aigner, 1973, DiTraglia and García-Jimeno, 2020).^29^ IV estimates reported in the seventh and eighth rows of Table 4 show that the naive IV estimator generates significantly inflated estimates compared to the GMM point-estimates of $E\left(X|T^{*}\right)$. As expected, we find that the IV estimator that uses $T^{R}$ as a proxy and $T^{G}$ as instrument (i.e., $\beta_{IV}^{R}$) is mostly similar to the GMM point-estimates of $E\left(X|T^{*}\right)$. As mentioned above, in the absence of misclassification in $T^{R}$ (i.e., when $\alpha_{0}+\alpha_{1}=0$), $\beta_{IV}^{R}$ is expected to be identical with $E\left(X|T^{*}\right)$. In our case, given the estimated misreporting rates (i.e., $\alpha_{0}+\alpha_{1}$ reported in the fifth row of Table 4), we would expect $E\left(X|T^{*}\right)$ to be: $\beta_{IV}^{R}\times \left[1-\alpha_{0}-\alpha_{1}\right]$ as long as $T^{G}$ is a valid instrument. However, we find that the IV estimator that uses $T^{G}$ as a proxy and $T^{R}$ as instrument (i.e., $\beta_{IV}^{G}$) is up to four times larger in magnitude compared to the GMM point-estimates of $E\left(X|T^{*}\right)$. This is expected given the high misclassification rate in $T^{G}$ in our data. That is, given the estimated misperception rate in our data (i.e., $\kappa_{0}+\kappa_{1}$ reported in the sixth row of Table 4), we would expect $E\left(X|T^{*}\right)$ to be: $\beta_{IV}^{G}\times \left[1-\kappa_{0}-\kappa_{1}\right]$ as long as $T^{R}$ is a valid instrument.

**Table 4:** Point identification results.

Estimation strategy	Fertilizer		Herbicide	
	Dummy	Kg/ha	Dummy	Lit/ha
$E\left(X|T^{*}\right)$: GMM	0.145***	0.742***	0.450***	0.925***
	(0.042)	(0.216)	(0.050)	(0.15)
$E\left(X|T^{R}\right)$: OLS	0.113***	0.677***	0.382***	0.799***
	(0.023)	(0.112)	(0.027)	(0.080)
$E\left(X|T^{G}\right)$: OLS	0.047**	0.224**	0.145***	0.284***
	(0.023)	(0.113)	(0.03))	(0.088)

$E\left(X|T^{R}\right)-E\left(X|T^{G}\right)$	0.066***	0.453***	0.237***	0.515***
	(0.026)	(0.133)	(0.033)	(0.094)

Estimated misclassification rates in $T^{R}$ and $T^{G}$				

Misreporting rates ($\alpha_{0}+\alpha_{1}$)	0.156	0.098	0.155*	0.114
	(0.233)	(0.221)	(0.081)	(0.139)
Misperception rates ($\kappa_{0}+\kappa_{1}$)	0.703***	0.745***	0.728***	0.733***
	(0.068)	(0.064)	(0.033)	(0.041)

Additional estimation results				

$\beta_{IV}^{R}$: 2SLS	0.172*	0.824*	0.532***	1.04***
	(0.088)	(0.422)	(0.103)	(0.312)
$\beta_{IV}^{G}$: 2SLS	0.488***	2.93***	1.65***	3.46***
	(0.108)	(0.549)	(0.187))	(0.445)
($T^{G}=T^{R}$) sample: OLS	0.115***	0.690***	0.398***	0.802***
	(0.031)	(0.143)	(0.036)	(0.113)

No. observation	3933	3933	3933	3933

*Note.* Standard errors clustered at enumeration area level are reported in parentheses. * p<0.10, ** p<0.05, *** p<0.01. For input use at the intensive margin, we employ inverse hyperbolic sine (IHS) transformation to keep observations with zero input use. The $T^{G}=T^{R}$ sample has 2504 observations.

### 5.3 Implications for input use behavior

In this section, we explore the relationship between the different forms of variety misperceptions and farmers’ observed input use behavior since farmers are expected to act up on their perceptions (wrong or right). To quantify and compare the behavioral implication of the different forms of misperception, we categorize farmer perceptions of improved varieties into the following four groups following Eq. (16) in Section 4: (i) correct improved variety perception (i.e., $\left(T^{G}=1;T^{R}=1\right)$ ≡ $V_{0}$); (ii) false positive perception (i.e., $\left(T^{G}=0;T^{R}=1\right)$ ≡ $V_{1}$); (iii) false negative perception (i.e., $\left(T^{G}=1;T^{R}=0\right)$ ≡ $V_{2}$) and (iv) correct local variety perception (i.e., $\left(T^{G}=0;T^{R}=0\right)$ ≡ $V_{3}$). Our estimates on farmers actual input use and intensification behavior are reported in Table 5. For each input, we report both OLS and FE estimates from Eq. (17) using correctly identified local variety ($V_{3}$) as a reference group. Furthermore, at the bottom of Table 5, we also report parameter equality tests. These tests probe the correlation between the different forms of variety perceptions and observed input allocation decisions based on the equality of the estimated coefficients conditional on the different forms of farmer perceptions (i.e., $V_{0};V_{1};V_{2};V_{3}$).

The estimates reported in Table 5 show that farmers are more likely to use fertilizer and herbicide inputs when they perceive growing an improved variety, irrespective of the true genetic quality of the cassava varieties they grow. The reverse holds true when farmers perceive growing unimproved variety. As such, farmers are more likely to crowd-in(out) fertilizer and herbicide inputs when they perceive the cassava varieties they planted are improved (unimproved), implying farmer variety perceptions affect their adoption decision of complementarity farm inputs. Reassuringly, the above findings on input use patterns are consistent across the intensive margins of fertilizer and herbicide input use, implying farmers are not just crowding-in(out) fertilizer and herbicide inputs but they are also changing their intensification behavior. The parameter equality tests reported at the bottom of Table 5 provides additional behaviorally relevant information about farmers’ input use and intensification choices. In particular, while we can consistently reject the null of $E\left(X|V_{0}\right)=E\left(X|V_{2}\right)$ and $E\left(X|V_{1}\right)=E\left(X|V_{2}\right)$, we mostly fail to reject the null of $E\left(X|V_{0}\right)=E\left(X|V_{1}\right)$. As shown in our analytical exercise, such input use behavior is expected to occur when misperception is the dominant source of variety misclassification (i.e., misperception reflects the underlying input use behavior of farmers).

The above results broadly corroborate the findings by Wineman et al., 2020, Emerick et al., 2016, Bulte et al., 2014. In the context of Tanzania, Wineman et al. (2020) report that farmers apply more fertilizer when they perceive growing an improved maize variety. Emerick et al. (2016) find evidence of crowding in of fertilizer and other modern cultivation practices among adopters of improved rice varieties in India. Similarly, in Tanzania (Bulte et al., 2014) find significant difference in farmers’ effort based on their perception about the improvement status of the cowpea varieties they planted. Our results have important implications in terms of identifying whether existing input complementarity estimates are driven by simple farmer perceptions or agronomic synergies. Our findings suggest that the allocation of fertilizer and herbicide inputs by farmers, which are complementary inputs to improved seed, are driven by their perceptions (wrong or right) instead of the true genetic qualities of cassava varieties they planted. This type of input use behavior may introduce inefficiency in the production process. This calls into question whether existing packaging and recommendations for complimentary agricultural inputs and other productivity-enhancing inputs can improve the technical efficiency of smallholders in the presence of pervasive input market imperfections (Abay et al., 2018).

**Table 5:** Misperception and input use behavior.

Panel A: Fertilizer use behavior								
	Dummy				Kg/ha			
	OLS	FE	OLS	FE	OLS	FE	OLS	FE
$\phi_{0}\left(V_{0}\right)$	0.115***	0.125***	0.106***	0.123***	0.690***	0.780***	0.636***	0.769***
	(0.031)	(0.043)	(0.031)	(0.043)	(0.143)	(0.210)	(0.142)	(0.21)
$\phi_{1}\left(V_{1}\right)$	0.057	0.105*	0.059***	0.103*	0.572***	0.828***	0.579***	0.819***
	(0.041)	(0.061)	(0.04)	(0.061)	(0.217)	(0.297)	(0.215)	(0.297)
$\phi_{2}\left(V_{2}\right)$	−0.016	−0.03	−0.027	−0.03	−0.018	−0.092	−0.074	−0.096
	(0.029)	(0.043)	(0.029)	(0.043)	(0.127)	(0.192)	(0.129)	(0.19)

$\phi_{0}=\phi_{1}$(p- value)	0.128	0.686	0.210	0.688	0.568	0.832	0.778	0.823
$\phi_{0}=\phi_{2}$(p- value)	0.000	0.000	0.000	0.000	0.000	0.000	0.000	0.000
$\phi_{1}=\phi_{2}$(p- value)	0.06	0.032	0.024	0.032	0.005	0.002	0.002	0.002

Panel B: Herbicide use behavior								

$\phi_{0}\left(V_{0}\right)$	0.398***	0.298***	0.265***	0.296***	0.802***	0.440***	0.624***	0.428**
	(0.036)	(0.059)	(0.036)	(0.058)	(0.113)	(0.172)	(0.111)	(0.171)
$\phi_{1}\left(V_{1}\right)$	0.289***	0.276***	0.204***	0.274***	0.540***	0.426**	0.401***	0.417**
	(0.051)	(0.062)	(0.053)	(0.062)	(0.143)	(0.193)	(0.144)	(0.192)
$\phi_{2}\left(V_{2}\right)$	−0.009	−0.003	−0.009	−0.004	−0.084	−0.124	−0.081	−0.130
	(0.034)	(0.051)	(0.033)	(0.051)	(0.113)	(0.17)	(0.110)	(0.170)

$\phi_{0}=\phi_{1}$(p- value)	0.020	0.477	0.040	0.492	0.003	0.906	0.067	0.928
$\phi_{0}=\phi_{2}$(p- value)	0.000	0.000	0.000	0.000	0.000	0.002	0.000	0.002
$\phi_{1}=\phi_{2}$(p- value)	0.000	0.000	0.000	0.000	0.000	0.007	0.000	0.007

Other controls	No	No	Yes	Yes	No	No	Yes	Yes
No. observation	3933	3933	3933	3933	3933	3933	3933	3933

*Note.* Standard errors clustered at enumeration area level are reported in parentheses. * p<0.10, ** p<0.05, *** p<0.01. For input use at the intensive margin, we employ inverse hyperbolic sine (IHS) transformation to keep observations with zero input use. The additional controls in the conditional regressions include household and plot level characteristics listed in Table 1.

### 5.4 Misperception and productivity

In this section, we explore the inferential implication of misperception focusing on land productivity. Following our analytical framework, we start our analysis by carrying out a hypothesis test to identify the relevant non-differential misperception assumption for yield as a first step model specification test. Table 6 reports the results of our non-parametric and parametric tests. In the first column of Table 6, the unconditional non-differential misperception assumption is rejected, implying that conditional on $T^{G}$, $T^{R}$ is informative about the conditional mean of Y. We then probe the non-differential misperception assumption conditioning on fertilizer and herbicide inputs, both at the extensive and intensive margins in the second and third column of Table 6, respectively. After conditioning on X, $T^{R}$ is not informative about the conditional mean of yield. In Panel B, we provide similar test results under parametric specifications. In the first column, without controlling for X, the coefficient associated with $T^{R}$ is highly significant, implying conditional on $T^{G}$, $T^{R}$ provides relevant information about the conditional mean of yield. In the second column, where we control for input use at the extensive margin, the coefficient associated with $T^{R}$ remains significant but the effect size declines considerably. However, in the third column, where we control for input use at the intensive margin, the coefficient associated with $T^{R}$ becomes insignificant. These test results imply the reduced form biophysical relationship between adoption of improved cassava varieties and yield can be estimated consistently using $T^{G}$ after conditioning on X.^30^

Following the above test results, we then examine the inferential implication of misperception focusing on land productivity. As discussed in Section 2.4, in the presence of variety misperception, the traditional regression specifications via $T^{R}$ or $T^{G}$ may generate biased parameter estimates. In an attempt to highlight the inferential challenges of variety misperception, we estimate the reduced form biophysical relationship between improved genetics and yield with and without accounting for farmers’ ex-post input choices (X). Our estimation results are reported in Panel A and B of Table 7. In each Panel, the first and second columns report unconditional OLS and fixed effect estimates. We then report both OLS and fixed effect estimates controlling for farmers ex-post input choices (X) in the third and fourth columns, respectively. In the final two columns, we report OLS and fixed effect estimates controlling for X and additional farmer and plot level characteristics. As shown in Panel B of Table 7, the size and sign of the expected bias due to variety misclassification is inferred by comparing the estimated coefficients associated with $T^{G}$ and $T^{R}$ (i.e., ${\overset{ˆ}{\gamma }}^{G}-{\overset{ˆ}{\gamma }}^{R}$).

**Table 6:** Non-parametric and parametric test results for yield.

Panel A: Non-parametric test results			
	$E\left(Y|T^{G},T^{R}\right)=E\left(Y|T^{G}\right)$	$E\left(Y|T^{G},T^{R},X\right)=E\left(Y|T^{G},X\right)$	
	Unconditional	X (dummy)	X (continuous)
p(CvM < CvM*)	0.000	0.444	0.476
p(KS < KS${}^{*}$)	0.000	0.172	0.184

(# rep)bootstrap	500	500	500

Panel B: Parametric test results			

$T^{G}$	0.292***	0.286***	0.288***
	(0.031)	(0.031)	(0.032)
$T^{R}$	0.098***	0.061**	0.035
	(0.031)	(0.031)	(0.031)

No. observation	3933	3933	3933

*Note.* For parametric tests, standard errors clustered at enumeration area level are reported in parentheses. * p<0.10, ** p<0.05, *** p<0.01. For input use at the intensive margin, we employ inverse hyperbolic sine (IHS) transformation to keep observations with zero input use. The dependent variable is log-transformed cassava yield.

Consistent with our analytical predictions, the naïve regression specifications based on $T^{R}$ generate parameter estimates that are biased towards zero compared to estimates based on $T^{G}$. The parameter equality test reported in Panel B of Table 7 (i.e., ${\overset{ˆ}{\gamma }}^{G}-{\overset{ˆ}{\gamma }}^{R}$) further confirms our prediction as the difference between the two conditional means is statistically significant.^31^

**Table 7:** Misperceptions and cassava yield.

Panel A: Using farmer reports ($T^{R}$)						
	OLS	FE	OLS	FE	OLS	FE
${\overset{ˆ}{\gamma }}^{R}$	0.165***	0.287***	0.096**	0.207***	0.096***	0.204***
	(0.031)	(0.069)	(0.029)	(0.065)	(0.031)	(0.065)

Panel B: Using the genetic test results ($T^{G}$)						

${\overset{ˆ}{\gamma }}^{G}$	0.319***	0.399***	0.294***	0.389***	0.294***	0.388***
	(0.031)	(0.049)	(0.03)	(0.048)	(0.03)	(0.048)

${\overset{ˆ}{\gamma }}^{G}-{\overset{ˆ}{\gamma }}^{R}$	0.154***	0.112**	0.197***	0.182***	0.198***	0.184***
	(0.36)	(0.046)	(0.036)	(0.051)	(0.048)	(0.057)

Inputs (Kg/ha)	No	No	Yes	Yes	Yes	Yes
Other controls	No	No	No	No	Yes	Yes
No. observation	3933	3933	3933	3933	3933	3933

*Note.* Standard errors clustered at enumeration area level are reported in parentheses. For input use at the intensive margin, we employ inverse hyperbolic sine (IHS) transformation to keep observations with zero input use. All our estimations control for those household and plot characteristics listed in Table 1. * p<0.10, ** p<0.05, *** p<0.01.

Next, we consider whether variety misperception induced input crowding-in and crowding-out behavior has any bearing on productivity. A central question related to this is how different the overall returns to improved genetics are with and without variety misperception. In Table 8, we report estimates from our regression specifications that explicitly quantify the correlation between the different forms of cassava variety perceptions and land productivity (i.e., cassava yield). Based on the estimates reported in Table 5, farmers’ input allocation decision on improved varieties and unimproved varieties that are incorrectly perceived as improved is statistically identical. Similarly, farmers’ input allocation decision on unimproved varieties and improved varieties that are incorrectly perceived as unimproved is also statistically identical. Estimates reported in the first four columns of Table 8 suggest that while input allocations are more responsive to farmer perceptions (right or wrong), yield responses to these input adjustments depend on the true genetic quality of the cassava varieties grown by farmers. For instance, the returns associated with cassava varieties that are incorrectly perceived as unimproved is lower than that of varieties that are correctly perceived as improved (i.e., $E\left(Y\left|V_{2}\right)<E\left(Y\right|V_{0}\right)$) but higher than the returns associated with varieties that are correctly perceived as unimproved (i.e., $E\left(Y\left|V_{2}\right)>E\left(Y\right|V_{3}\right)$). Moreover, the returns associated with improved varieties that are incorrectly perceived as unimproved is still higher than that of unimproved varieties that are incorrectly perceived as improved (i.e., $E\left(Y|V_{2}\right)>E\text{(}Y|V_{1}$)). In fact, conditional on observed input choices, farmer perceptions become irrelevant as the conditional mean of Y only varies with the genetic quality of the cassava varieties grown by farmers (i.e., whether it is improved or not). In particular, after conditioning on X, we consistently fail to reject the null of $E\left(Y\left|V_{0}\right)=E\left(Y\right|V_{2}\right)$ and $E\left(Y\left|V_{1}\right)=E\left(Y\right|V_{3}\right)$.

To understand the mechanisms through which variety misperception may affect farmers actual production efficiency, we next explore heterogeneity in the yield responses to inputs by interacting farmers observed fertilizer and herbicide input allocations with the different forms of variety perceptions. We do so since the different forms of variety perceptions affect the allocation of fertilizer and herbicide inputs, which are complementary inputs to improved seed. In particular, in the presence of variety misperception, input allocation is expected to be based on $T^{R}\equiv T^{*}$, which is different from an allocation based on $T^{G}$. Hence, farmers holding false positive and negative variety perceptions base their input allocation decisions on the wrong variety type, which is likely to be inefficient. Our estimates reported in the last two columns of Table 8 clearly show that the association between input use and yield is weaker in the presence of variety misperception in the sense that the yield response of fertilizer and herbicide inputs is higher when there is no misperception. For instance, OLS estimates reported in the fifth column of Table 8 show that fertilizer input is yield-enhancing even when farmers hold false positive variety perception but not at the rate that would have been achieved with correct variety perception (i.e., while the interaction term between $V_{0}$ and fertilizer input is 0.056, the interaction term between $V_{1}$ and fertilizer is only 0.038.) In addition, while the interaction term between $V_{0}$ and fertilizer input is statistically significant both in the OLS and FE specifications, the interaction term between $V_{1}$ and fertilizer input become statistically insignificant in the FE specification.

**Table 8:** Misperceptions and input productivity.

Dependent variable: Log cassava yield						
	OLS	FE	OLS	FE	OLS	FE
Correct improved ($V_{0}$)	0.375***	0.570***	0.301***	0.492***	0.169***	0.332***
	(0.041)	(0.07)	(0.039)	(0.067)	(0.049)	(0.085)
False positives ($V_{1}$)	0.028	0.189**	−0.018	0121	−0.089	0.012
	(0.054)	(0.083)	(0.057)	(0.082)	(0.076)	(0.110)
False negatives ($V_{2}$)	0.247***	0.348***	0.254***	0.363***	0.227***	0.394***
	(0.044)	(0.066)	(0.042)	(0.062)	(0.050)	(0.073)
Fertilizer (Kg/ha)			0.07***	0.048***	0.032**	0.031
			(0.006)	(0.012)	(0.014)	(0.021)
Herbicide (lit/ha)			0.03***	0.064***	−0.006	0.059**
			(0.009)	(0.015)	(0.017)	(0.028)
Correct improved × Fertilizer					0.056***	0.044*
					(0.017)	(0.026)
False positives × Fertilizer					0.038*	−0.001
					(0.022)	(0.033)
False negatives × Fertilizer					0.013	0.007
					(0.019)	(0.024)
Correct improved × Herbicide					0.064***	0.043
					(0.022)	(0.036)
False positives × Herbicide					0.039	0.064
					(0.035)	(0.047)
False negatives × Herbicide					0.015	−0.040
					(0.0245)	(0.037)

$E\left(Y|V_{0}\right)=E\left(Y|V_{1}\right)$(p- value)	0.000	0.000	0.000	0.000	0.000	0.001
$E\left(Y|V_{0}\right)=E\left(Y|V_{2}\right)$(p- value)	0.000	0.006	0.137	0.083	0.216	0.475
$E\left(Y|V_{1}\right)=E\left(Y|V_{2}\right)$(p- value)	0.000	0.095	0.000	0.011	0.000	0.000

Inputs (X)	No	No	Yes	Yes	Yes	Yes
Other controls	No	No	Yes	Yes	Yes	Yes
No. observation	3933	3933	3933	3933	3933	3933

*Note.* Standard errors clustered at enumeration area level are reported in parentheses. * p<0.10, ** p<0.05, *** p<0.01. The dependent variable is log-transformed cassava yield. Base category: Correctly identified local varieties $\left(V_{3}\right)$. For input use at the intensive margin, we employ inverse hyperbolic sine (IHS) transformation to keep observations with zero input use. The additional controls include household and plot characteristics listed in Table 1.

The results reported in Table 5, Table 7, Table 8 also (indirectly) highlight the opportunity cost of misperceptions. In particular, given our results, we can reasonably argue that while input allocations are more responsive to farmer perceptions (right or wrong), yield responses to these input adjustments depend on the true genetic quality of the cassava varieties grown by farmers. Intuitively, this implies that variety misperception may introduce inefficiencies in the production process by distorting the allocation of complementary inputs to improved seed relative to their allocation with correct variety perception (i.e., irrespective of its optimality). For instance, farmers holding false positive variety perception could have reduced their input use without offsetting their yield by a significant margin had they acted upon perfect information about the genetic quality of the cassava varieties they planted (i.e., by crowding-out fertilizer and herbicide inputs). On the other hand, farmers holding false negative variety perception could have increased their cassava yield had they acted upon perfect information about the genetic quality of the cassava varieties they planted (i.e., by crowding-in more fertilizer). As such, rectifying misperception may induce efficiency-enhancing reallocation of fertilizer and herbicide inputs by farmers.^32^ When quality is difficult to observe, economic theory suggests that intervention such as regulatory systems that impose quality standards could play a vital role in improving overall market efficiency (Maredia et al., 2019, Gilligan and Karachiwalla, 2019). For instance, Gilligan and Karachiwalla (2019) show that an input assurance scheme in Uganda influences farmers’ beliefs regarding the quality of inputs (i.e., herbicide and hybrid maize seeds) as well as their adoption patterns. As such, addressing agricultural input and seed market frictions may improve farmers’ investment choices and productivity outcomes.

## 6 Conclusions

This paper illustrates, both analytically and empirically, the inferential and behavioral implications of misperceiving and misreporting crop varieties using a unique self-reported and genetic test based cassava variety identification data from Nigeria. By examining the conditional means of input use functions non-parametrically, we identify and decompose the origins of variety misclassification into misreporting and misperception. In particular, we show that crop variety misclassification in our data is mostly driven by misperceptions. A key implication of this result is that misperception reflects the underlying decision making process of farmers and hence should not be regarded as misreporting or survey error *per se* as it provides behaviorally relevant information about farmers’ input use and intensification choices. Our findings have important implications both in terms of improving statistical inference and understanding the potential costs of input market frictions. In terms of inference, we characterize the expected bias under misperception and misreporting and show the implications of treating misperception as misreporting and vice versa. We also show that crop variety misperception induces crowding-in(out) of fertilizer and herbicide inputs. However, our estimates on the yield responses of these inputs suggest that misperception induced input allocation may not necessarily be yield-enhancing. As such, our results underscore the potential costs of agricultural input and seed market imperfections and hence addressing agricultural input and seed market frictions can play an important role in improving farmers’ investment choices and productivity outcomes.

In terms of future research agenda, several important extensions can be proposed on the basis of our results. First, understanding the existence and persistence of misperception and the role of potential remedies such as access to quality information, verification and regulatory mechanisms will be important to address agricultural input market frictions. For instance, Gilligan and Karachiwalla (2019) show that an input assurance scheme in Uganda changes farmers’ beliefs regarding the quality of inputs as well as their adoption patterns. Second, more accurate diagnosis of farmers input use and production choices will require improved measurement of crop variety traits, input and production data. This implies increased investments in improving data quality by national governments and their development partners. In the short term, such investments may focus on identifying and mainstreaming methodological innovations and traceability systems (e.g., DNA-fingerprinting for monitoring varietal adoption) into large scale national surveys.^33^ In terms of data collection efforts and strategies, we argue that the choice of the most appropriate variety data collection can be studied as an optimal survey design problem.

Third, our results are only as credible as the key identification assumptions we use to derive our estimates. In particular, our main non-parametric tests for identifying the presence of misclassification are applied in the context of an exogenous regressor subject to non-differential misclassification. Hence our results might not hold if the misclassification process in the two proxies is differential. Therefore, it would be useful to explore approaches for identifying and decomposing misclassification into misreporting and misperception in the presence of differential (i.e., endogenous) misclassification. Our estimates can also be sensitive to possible nonclassical measurement error in input and production data (Abay et al., 2019). In particular, since we relied on self-reported input and production data, our estimates can be biased in the presence of correlated measurement error in crop variety, input and production data. As such, combining innovative data collection approaches in research designs that allow for causal inference can help confirm our findings and provide additional insights on the inferential and behavioral implication of misperception.

## CRediT authorship contribution statement

**Tesfamicheal Wossen:** Conceptualization, Data curation, Formal analysis, Investigation, Methodology, Writing – original draft, Writing – review & editing. **Kibrom A. Abay:** Conceptualization, Investigation, Methodology, Writing – original draft, Writing – review & editing. **Tahirou Abdoulaye:** Conceptualization, Funding acquisition, Project administration, Writing – review & editing.

## Declaration of Competing Interest

The authors declare that they have no known competing financial interests or personal relationships that could have appeared to influence the work reported in this paper.

## Footnotes

1 The correlation between the true value and its measurement error is always negative.

2 As discussed above, in our context misperception arises when farmers’ latent variety perceptions are identical with their reports but not with the genetic test results. On the other hand, misreporting arises when farmers’ latent variety perceptions are identical with the genetic test results but not with their reports.

3 Note that, our empirical specifications include a constant term to ensure that $E\left({ɛ}^{*}\right)=0$.

4 It is straightforward to show that $Cov\left(T^{R},T^{*}\right)=Var\left(T^{*}\right)\left[1-\alpha_{0}-\alpha_{1}\right]$. Moreover, since, the variance of $T^{*}$ and $T^{R}$ is given by $p^{*}\left(1-p^{*}\right)$ and p(1−p), respectively, the variance of $T^{*}$ can be expressed as: $\frac{\left(p-\alpha_{0}\right)\left(1-p-\alpha_{1}\right)}{{\left(1-\alpha_{0}-\alpha_{1}\right)}^{2}}$.

5 The researcher rules out market frictions such as variety misperceptions by farmers due to asymmetric quality information in the local markets.

6 Notice that, our exclusion restriction relies on the weaker conditional mean independence assumption instead of the stronger conditional independence assumption (i.e., $X⊥\left(T^{G},T^{R}\right)|T^{*}$).

7 Also, it is not nested w.r.t the test $T^{*}=T^{G}$.

8 In fact in Eq. (7), $P\left(T^{R}=T^{*}\right)=1$ implies $\beta^{G}=0$ so long as $E\left({ɛ}^{*}T^{*}\right)=0$ in Eq. (1), $E\left({ɛ}^{RG}T^{G}\right)=0$ and $T^{G}$ has strong correlation with $T^{*}$.

9 Also note that, our formal non-parametric test relies on the conditional mean independence assumption. Hence no misclassification implies misclassification (if any) will not distort the conditional mean of the input use function. However, this may not hold for other moments of the conditional input use distribution (Wilhelm, 2019, Mahajan, 2006).

10 For more details, see Mahajan (2006) Appendix A.1.

11 Note that, given Assumption 2.5, we cannot test whether $T^{*}=T^{R}$ since both sides are equal.

12 Note that, unless $T^{G}$ is exogenous in Eq. (9), $\gamma^{G}$ will not represent the causal effect of $T^{G}$ on Y. We maintain this assumption since our main focus is to overcome potential bias due to measurement error instead of the endogeneity of the adoption decision.

13 In particular, we have: $Cov\left(X,\mu^{R}\right)=-\left(\alpha_{0}+\alpha_{1}\right)Cov\left(X,T^{G}\right)$.

14 Note that, without imposing the non-differential measurement error assumption, $T^{R}$ cannot be used as an additional conditioning variable in our regressions since $T^{R}$ is endogenous. Although the case of differential misperception is beyond the scope of this paper, identification can be achieved via an exclusion restriction for misperception (Nguimkeu et al., 2019, Yanagi, 2019).

15 Still, identification and consistent estimation of the biophysical relationship between $T^{G}$ and Y via Eq. (12) relies on the assumption that $T^{G}$ is measured without error. As an additional robustness check, we have also attempted to relax this assumption. In particular, following a similar logic as in Section 2.3, we assume that there exists a latent but unobserved variable, $W^{*}$, which captures both genetic quality and farmer perceptions consistently. Note, however, that $W^{*}\neq T^{*}$ unless $T^{G}=T^{*}$, in which case $\gamma^{G}\equiv E\left(Y|W^{*}\right)$ could have been estimated consistently via Eq. (12). Under this scenario, we assume the following non-differential measurement error assumption for yield: $E\left(Y\left|W^{*},T^{G},T^{R},X\right)=E\left(Y\right|W^{*},X\right)$. Hence, we maintain the same identification conditions described in Section 2.3 to point-identify $E\left(Y|W^{*}\right)$, except that the latent variable, $T^{*}$, in 2.4(i)-(iii) is replaced by $W^{*}$ and $T^{R}$ serves as ILV to deal with the measurement error in $T^{G}$ (i.e., in our context, it is reasonable to assume that genotyping error, if any, is likely to be small).

16 Nigeria is the largest producer of cassava in the world, contributing roughly 20% of the global cassava production (Wossen et al., 2019b). The crop is largely produced by farm households as an important source of basic nutrition and income (Wossen et al., 2019a). It is tolerant to drought and can grow across a range of agro-climatic conditions and poor soils. Due to its resilience, it is traditionally viewed as a food security crop and plays a key role in farmers crop diversification strategies.

17 Among others, we collected detail cassava variety related information using the following questions: (a) how many cassava varieties do you grow on this plot; (b) list the names of the varieties grown in this plot (local names as given by farmers) and (c) is the variety local or improved.

18 The DNA-fingerprinting analysis was led by the Genomic Diversity Facility (GDF) of Cornell University.

19 Since production and input use information were collected at the plot instead of variety level, our analysis is restricted to homogeneous plots. In addition, to ensure that farmers’ input use behavior reflects their input allocation decision for cassava, intercropped plots where cassava is not the major crop were also excluded.

20 $P\left\{T^{G}=0|T^{R}=1\right\}$ and $P\left\{T^{G}=1|T^{R}=0\right\}$ rates in our data are 19.6% and 57.3%, respectively. On the other hand, $P\left\{T^{R}=0|T^{G}=1\right\}$ and $P\left\{T^{R}=1|T^{G}=0\right\}$ rates in our data are 36.22% and 36.6%, respectively.

21 As discussed before, due to the informality of the underlying cassava seed market, farmers may also acquire unknown varieties due to unregulated stem disseminations as part of large-scale input subsidy schemes. Misperception or misreporting could also arise due to low awareness and inadequate technical information to fully understand and identify key variety traits. For instance, farmers may perceive older varieties that had been recycled for several years as unimproved although these varieties were officially released after rigorous testing (Wineman et al., 2020, Kosmowski et al., 2016, Floro et al., 2018). Finally, misreporting could arise due to survey errors (e.g., recording errors, errors by enumerators, intentional misreporting by farmers, etc.). For instance, farmers may intentionally report improved varieties as unimproved if they anticipate returns to lying (e.g., they may believe that the survey team may facilitate access to recently released improved varieties).

22 This is chosen for simplicity, despite the fact that some of our outcomes, those involving extensive margin of input use, are binary.

23 Since, unobserved heterogeneity among farmers may correlate with farmers’ variety misperception, input crowding-in (out) decisions and yield outcomes, we control for household fixed effects.

24 These include the household and plot level characteristics listed in Table 1.

25 Note that, as discussed in Section 2.2, testing for the presence of misclassification in $T^{R}$ in Panel B of Table 3 is equivalent to testing for the exclusion restriction of $T^{G}$.

26 That is, the above test results do not directly affirm the absence of misclassification in $T^{R}$ entirely in the sense that the misclassification rates will indeed be zero at every points of the conditional input use distribution.

27 Note that, as argued in Abay et al. (2021) land is not a credence good per se but plot size misperception could arise due to imprecise plot size beliefs inferred from observable plot characteristics such as soil fertility, productivity etc. which vary across seasons and plot types. The source of misperception for land size could also be related to land market frictions and other plot size information signals.

28 Note that, our non-parametric test results do not directly affirm the absence of misclassification in $T^{R}$ entirely in the sense that the misclassification rates will indeed be zero at every points of the conditional input use distribution.

29 Note that, without the non-differential measurement error assumption, we cannot sign the bias in IV estimates.

30 This assumes $T^{G}$ is strictly exogenous.

31 As an additional robustness check, we have also attempted to point-identify $E\left(Y|W^{*}\right)$ via GMM based on the identification assumptions discussed in Section 2.4 as well as via OLS by discarding observations with conflicting $T^{G}$ and $T^{R}$ values. We find that while the GMM point-estimate of $E\left(Y|W^{*}\right)$ is 0.43, the OLS estimate of $E\left(Y|T^{G},X\right)$ from the restricted sample that discards observations with conflicting $T^{G}$ and $T^{R}$ values is 0.37. Note that, based on the non-parametric test results reported in Table 6, these conditional mean estimates are expected to be the same as the conditional mean estimates (i.e., $E\left(Y|T^{G},X\right)$) reported in Panel B of Table 7.

32 This conclusion is suggestive and is drawn based on our estimates on the average returns to fertilizer use given false negative and positive variety perceptions. In particular, our estimates of fertilizer use behavior in Table 5 and the average returns to fertilizer use in Table 8 suggest that it may be possible to reduce fertilizer use among farmers holding false positive variety perceptions without offsetting their productivity. Hence, given the observed fertilizer input use and cassava production in our data, our results suggest that it may be possible to improve current production levels by simply re-allocating fertilizer input across farmers holding false positive and false negative variety beliefs.

33 However, mainstreaming traceability systems into large scale surveys may have significant cost implication.
